# Adolescent and young adult sleep and sleep-related behaviour change before and during the COVID-19 pandemic lockdown in Canada

**DOI:** 10.1080/00049530.2024.2408019

**Published:** 2024-10-14

**Authors:** Nicole E. Carmona, Samlau Kutana, David Sumantry, Onkar Marway, Alison Carney, Maya Amestoy, Aleksandra Usyatynsky, Colleen E. Carney

**Affiliations:** Department of Psychology, Toronto Metropolitan University, Toronto, Canada

**Keywords:** Adolescents, young adults, sleep, self-management, mhealth, COVID-19

## Abstract

**Background:**

Sleep disturbance is common in adolescents and young adults (AYAs), impacted by stress and academic/scheduling demands that conflict with biological phase delay. COVID-19 lockdowns allowed us to study sleep in AYAs when there are lessened scheduling demands. Additionally, we could test whether a sleep self-management app was helpful during lockdowns.

**Method:**

AYAs (ages 15–24 years) before (Pre-Lockdown; *n* = 65) and during lockdowns in Canada (Lockdown; *n* = 40) completed sleep diaries on the app; set goals based on generated feedback; and completed more sleep diaries pursuing whatever post-feedback goals they set.

**Results:**

The Lockdown group reported later and less variable rise times (RT) and spent more time in bed (TIB), both awake and asleep. Pre-Lockdown set a goal to reduce RT variability whereas Lockdown set a goal to decrease TIB, and AYAs made behaviour changes to meet their goals. For both groups, sleep onset, duration of awakenings, sleep duration and efficiency, and insomnia severity significantly improved at endpoint.

**Conclusions:**

AYAs slept differently during lockdowns, perhaps due to decreased scheduling. The pandemic revealed the need for accessible strategies to improve sleep health. Findings support the feasibility of using evidence-based apps, and that AYAs can effectively use self-management tools across variable global and social contexts to improve their sleep.

## Key Points

**What is already known about this topic:**

1. Adolescents and young adults (AYAs) experience myriad sleep problems that result from an interaction between (1) biological factors promoting later bedtimes while still requiring more time to recovery from sleep debt than adults, (2) environmental factors such as early school start times, and (3) behavioural factors that delay bedtime and increase pre-sleep arousal.
2. Surveys conducted during initial COVID-19 lockdowns indicated increased rates of sleep disturbance in AYAs.
3. AYAs unique sleep needs and low access to care indicate that tailored, digital self-management tools may be an optimal strategy to improve sleep health in this group.

**What this study adds:**

1. Using a transdiagnostic sleep app, we found that AYAs completing sleep diaries during lockdowns spent more time in bed resulting in more time asleep and more time awake, and their rise time shifted later and became more consistent.
2. AYAs before and during lockdowns set different goals to improve their sleep, but both groups used the app to set and make progress towards their sleep goals and experienced improvements in sleep parameters and subjective insomnia severity.
3. Our data provide initial support for the use of tailored digital solutions to reach this at-need group and indicate that AYAs flexibly use self-management tools to improve their sleep health under shifting global contexts.

## Introduction

Sleep disturbances are common among adolescents and young adults (AYAs) (Hicks et al., 2002; Hysing et al., 2013; Sivertsen et al., 2019). Studies have reported insomnia prevalence rates of 18.5% in adolescents and 30.5% in young adults using the Diagnostic and Statistical Manual of Mental Disorders, 5^th^ edition criteria (Hysing et al., 2013; Sivertsen et al., 2019). Rates of hypersomnia, whether alone or comorbid with insomnia, exceed 8% (Breslau et al., 1996; Roberts et al., 2001). Moreover, 31–62% of AYAs report poor sleep quality and many are dissatisfied with their sleep (Becker et al., 2018; Ghrouz et al., 2019; Hicks et al., 2002). Adolescence is a significant period of sleep-related changes: the accumulation of sleep pressure slows throughout the day, facilitating longer periods of time awake in a 24-hour period, but there is no change in the speed of recovery from sleep debt (Crowley et al., 2018). Despite an ability to stay awake later into the evening, adolescents still need more sleep than adults.

Environmental and behavioural factors present barriers to healthy sleep among AYAs that, together with biological changes, result in a “perfect storm” (Carskadon, 2011, 2002; Crowley et al., 2018; Hoyt et al., 2018). AYAs have constrained sleep schedules relating to early school start times, extracurricular activities, and periodically increased academic demands (Wheaton et al., 2016). This leads to insufficient sleep, early rise times despite biological tendencies towards eveningness, and pronounced daytime sleepiness amongst AYAs (Ming et al., 2011; Wheaton et al., 2016). AYAs also engage in behaviours that delay bedtime, contribute to sleep restriction and increase pre-sleep arousal, including evening technology use (e.g., gaming, social media), socialising, and use of caffeine and sleep-interfering substances without complete understanding of the risks or consequences (Cain & Gradisar, 2010; Calamaro et al., 2009; Carskadon, 2002; Knutson & Lauderdale, 2009). Furthermore, inconsistent bedtimes and rise times across weekdays and weekends cause symptoms similar to jet lag (“social jetlag”), negatively impacting daytime functioning and the regularity of sleepiness and alertness. Thus, AYAs have sleep needs and circadian changes that differ from other age groups and result in various sleep problems (e.g., insomnia, hypersomnia, circadian rhythm disorders), requiring a transdiagnostic sleep health approach (Harvey, 2016).

The COVID-19 lockdowns provided a unique opportunity to study unconstrained sleep among AYAs as they had the potential to either improve or worsen sleep for AYAs; whereas scheduling demands decreased, symptoms of self-reported sleep disturbance increased (Zhou et al., 2020). Increased schedule flexibility and reduced work demands may provide an ideal opportunity for sleep health-promotive interventions (Simpson & Manber, 2020), which AYAs often struggle to access (Boerner et al., 2015; Honaker & Meltzer, 2016; Koffel et al., 2020). Lockdowns may also provide the opportunity for positive behaviour changes among AYAs, such as adopting more chronotype-compatible schedules (e.g., eliminating morning commutes may allow for later rise times) and allowing for longer sleep (Becker & Gregory, 2020; Gruber et al., 2020; Simpson & Manber, 2020). Such changes can be particularly valuable for AYAs with preferences for later schedules who experience constrained sleep during the school week (Becker & Gregory, 2020; Crowley et al., 2018).

Alternatively, increases in sleep disturbance, sleep-related anxiety and COVID-19-related stress were postulated to negatively influence adolescent sleep (Becker & Gregory, 2020); similar findings were reported among adults (Simpson & Manber, 2020). Lockdowns may have produced negative behavioural changes. Decreases in schedule regularity due to lockdowns (e.g., in the timing of final awakening, meals, light exposure) may have worsened sleep. Increased opportunity for sleep/resting and decreased structure and physical activity throughout the day could negatively impact hypersomnia, excessive daytime sleepiness, or fatigue. Indeed, AYAs were less active and more sedentary during lockdowns (López-Bueno et al., 2020; Moore et al., 2020). Transitions to remote learning may have contributed to more time in bed, diffusing sleep drive and contributing to conditioned arousal (Becker & Gregory, 2020). At the time of submission, few countries are in stringent lockdown, but COVID-19 continues. It is important to understand the nature of AYA’s sleep during lockdowns and whether accessible intervention is possible; this helps us to prepare for future pandemic public health strategies.

We previously investigated the feasibility, acceptability, and efficacy of an AYA-co-designed sleep self-management app called Delivering Online Zzz’s with Empirical Support (DOZE) (Carmona et al., 2021). We conducted a second wave of data collection during the COVID-19 lockdown to test whether: 1) AYAs slept differently during lockdown, and 2) they used DOZE differently during this period (i.e., set different goals for behaviour change, seek out psychoeducation to support achieving their goals), when they may have more control over their schedules and routines. As lockdowns had the potential to impact AYA sleep both positively and negatively, specific hypotheses were not made and all analyses are exploratory.

## Materials and methods

### Participants

Individuals were eligible to participate if they were between 15 and 24 years old, reported sleep dissatisfaction, and were living in Canada. Demographic characteristics of study participants can be found in Table 1. The mean age was 20.54 years (*SD* = 2.53); 21.4% of Lockdown app users were adolescents (15–18) and 67.9% were young adults (19–24; missing data, *n* = 6, 10.7%). Most app users were female (62.5%) and European Canadian (71.4%).

**Table 1.** Demographic characteristics of app users before and during COVID-19 lockdowns.

		Group	
		Pre-Lockdown (*n* = 83)	Lockdown (*n* = 56)
Baseline Characteristics		*N* (%)	*N* (%)
Sex			
	Female	57 (68.7)	35 (62.5)
	Male	26 (31.3)	19 (33.9)
	Other	0 (0)	2 (3.6)
Ethnicity			
	Aboriginal Canadian	0 (0)	5 (8.9)
	Caribbean Canadian	1 (1.2)	0 (0)
	East/Southeast Asian Canadian	15 (18.1)	4 (7.1)
	European Canadian	37 (44.6)	40 (71.4)
	Latin/Central/South American Canadian	4 (4.8)	1 (1.8)
	South Asian Canadian	8 (9.6)	3 (5.4)
	West Asian/Arab Canadian	1 (1.2)	0 (0)
	Pacific Islander Canadian	1 (1.2)	0 (0)
	Other	16 (19)	3 (5.4)
Seeing HCP for sleep disorder^a^		4 (4.8)	4 (7.1)
Seeing HCP for mental disorder^a^		14 (16.9)	12 (21.4)
		*M* (SD)	*M* (SD)
Age		20.17 (2.49)	20.54 (2.53)
ISI		12.48 (4.67)	10.32 (5.11)

Means and standard deviations are presented for continuous variables while percentages are presented for categorical variables. Abbreviations: HCP = health care professional, ISI = Insomnia Severity Index.

^a^Reflects the number and percentage of participants answering “yes” to currently seeing an HCP for this issue.

We have previously described the inclusion criteria and recruitment strategies (Carmona et al., 2021; ClinicalTrials.gov NCT03960294). Recruitment for the “Pre-Lockdown” group took place from 3 September 2019 to 14 January 2020, and data collection was completed by 31 January 2020 (Carmona et al., 2021). Recruitment for the “Lockdown” group took place between 24 March 2020 to 8 May 2020, and data collection concluded by 10 June 2020. The Ontario provincial lockdown lasted from March 17-9 June 2020. During this time, a state of emergency was declared. Recreational programs, provincial parks, libraries, schools, daycares, religious settings, bars and restaurants, and non-essential businesses were ordered to close; social gatherings were prohibited; and federally, all non-essential travel was restricted (Nielsen, 2020). Although we did not collect data on participants’ educational and occupational status, the majority of participants (with the exception of essential workers) undoubtedly experienced significant changes to their school/work routines by participating in online schooling or working from home. All participants provided informed consent.

### Measures

#### DOZE

DOZE is a free sleep self-management app co-created by AYAs and healthcare providers based on the Transdiagnostic Sleep and Circadian Intervention (Harvey, 2016). We have previously described the app and its design process (Carmona et al., 2021). Briefly, we involved AYAs in an iterative, experienced-based, co-design process and designed the app based on their feedback and stated priorities for a sleep app, resulting in each of the core features described below. DOZE provides tailored, transdiagnostic, evidence-based behavioural sleep medicine strategies to address the diverse sleep problems AYAs experience (e.g., sleeping too little, sleeping too much, variable sleep schedules, use of sleep-interfering substances, fatigue). At the time of the study, DOZE was accessed through a web browser on any device. DOZE utilises a daily sleep diary that is completed for 2 weeks and assesses nightly sleep, naps, and use of sleep-interfering substances. Sleep indices are calculated based on the sleep diary, including bedtime and rise time variability, sleep onset latency (SOL), wake after sleep onset (WASO), morning lingering in bed, sleep efficiency (SE), total sleep time (TST), total wake time (TWT), and time in bed (TIB). Based on the sleep diary, DOZE provides personalised feedback as to whether each of these sleep indices are too high or low relative to age-adjusted norms (Paruthi et al., 2016). For instance, an individual with TST under 8 hours would receive feedback that the amount they are sleeping is too little. Following this feedback, users can set goals in the following areas: “Naps”, “Too much/too little time in bed”, “Sleep interfering substances”, “Lingering in bed in the morning”, “Sleepiness”, and “Jetlag without traveling”. Users are only provided the opportunity to set goals in areas that are relevant to their sleep patterns. For instance, a user who did not log any naps in the sleep diary would not be given the opportunity to set a goal to reduce napping. To help users meet their goals, DOZE provides interactive tips including quizzes and psychoeducation for help with difficulty winding down, being a night owl, difficulty rising in the morning, daytime sleepiness, and fatigue. Users can save goals and tips to their personal dashboard. After setting goals, AYAs use the sleep diary for 2 or more weeks and view their progress over time in their personal dashboard. In this study, participants used DOZE for a 2-week baseline period and for 2 weeks post-feedback.

#### Self-report questionnaires

Participants completed various self-report questionnaires assessing sleep and daytime functioning as part of a larger study. To address this study’s aims, we only report on the Insomnia Severity Index (ISI). The ISI (Morin, 1993) is a 7-item scale used to measure self-reported sleep disturbance and is considered the gold standard assessment of insomnia symptoms. Items are scored on a 5-point Likert scale from 0 (none) to 4 (severe). Total scores range from 0 to 28. The ISI has demonstrated good to excellent psychometric properties in clinical and community samples of adolescents and adults, including internal consistency, test-retest reliability, and convergent validity (Bastien et al., 2001; Chung et al., 2011; Marway et al., 2023; Morin et al., 2011). To distinguish good and poor sleepers, research supports a cut-off score of 10 for community samples of adults and a score of 9 for adolescents (Chung et al., 2011; Morin et al., 2011). Given the mean age of the sample, we elected to use a more conservative cut-off score of 10 to identify good and poor sleepers in this study. In this sample, internal consistency of the ISI was good at baseline (Cronbach’s ɑ = 0.82; *n* = 137) and acceptable at endpoint (Cronbach’s ɑ = 0.74; *n* = 77).

### Procedure

Interested AYAs were directed to the DOZE website (www.dozeapp.ca), which provided the consent form and privacy policy. After providing consent, participants completed a quiz to confirm understanding of key aspects of the consent form (study purpose, risks/benefits, limits of confidentiality) and ensure that consent was informed. A score of 100% was required to proceed, ensuring that all participants provided informed consent. Participants completed a demographic form and all self-report questionnaires online. Participants then received a link to access DOZE. All participants were instructed to use DOZE for 2 weeks (baseline) to complete daily sleep diaries. After the baseline, they received personalised feedback and had the opportunity to set goals, access tips, and complete 2 more weeks of sleep diaries (post-feedback). The study coordinator contacted participants via email to maintain adherence when several consecutive days of sleep diaries were missed. After 4 weeks of using DOZE, participants completed the endpoint self-report questionnaires online. Participants received an honorarium for participating in each component of the study. The study was approved by the Research Ethics Board at Toronto Metropolitan University (REB #2019-184).

### Statistical analysis

Demographic data were analysed descriptively. Independent samples *t*-tests and chi-square analyses were used to compare the baseline characteristics between Lockdown completers and dropouts, and Lockdown vs. Pre-Lockdown completers to rule out the influence of these variables on app use. Variables were inspected for normality and extreme outliers using boxplots and histograms. Non-normal variables were log(10) or square root transformed and re-inspected, upon which all were sufficiently normal for parametric analyses.

To address our first aim, we compared baseline sleep in Pre-Lockdown and Lockdown participants among all app users who completed sleep diaries for 7 or more days using Welch’s two-sample t-tests. We used the Benjamini-Hochberg False Discovery Rate (.05) to adjust for multiple comparisons. To address our second aim, we first used chi-square analyses to compare goal setting and tip use between Lockdown and Pre-Lockdown participants. Next, we examined goal-related behaviour change (derived from sleep diaries; e.g., limiting TIB, reducing jet lag) from baseline to post-feedback using linear mixed models, including group (Lockdown, Pre-Lockdown) as the between-persons factor and time (baseline, post-feedback) as the within-persons factor, and estimated using restricted maximum likelihood. Significant interactions were followed-up with simple effects analyses. Lastly, we used linear mixed models to explore whether app use and goal setting was associated with differences in sleep symptoms and schedules before and during lockdowns among participants who completed 2 weeks of sleep diaries at baseline and post-feedback.

## Results

### User characteristics

**Figure 1.**
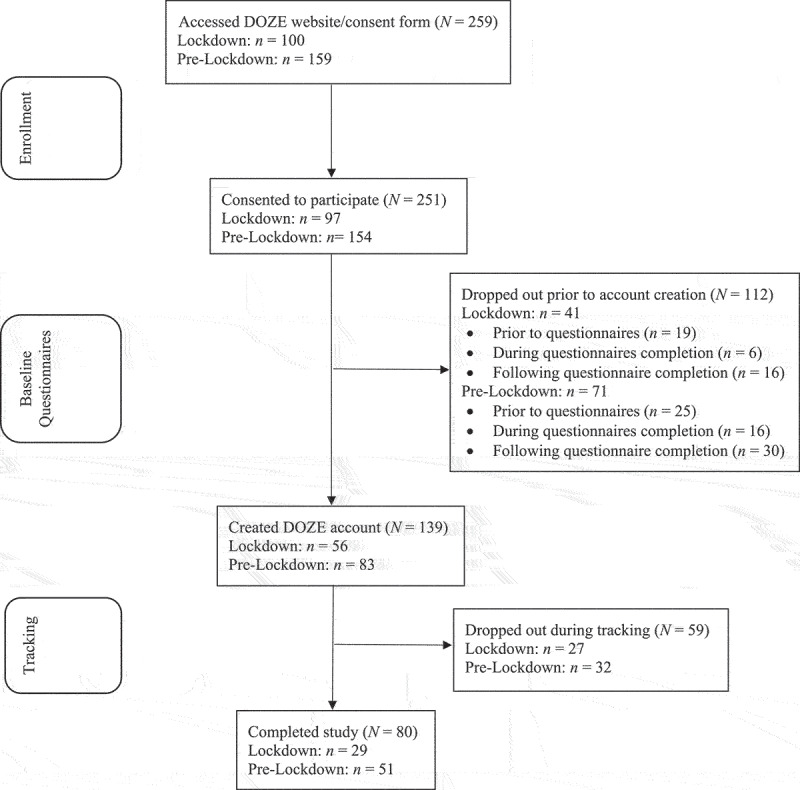
Flowchart of study participants.

Participant flow is illustrated in Figure 1. Of those who consented to participate during Lockdown, 58% created an app account (*n* = 56; “app users”), and 52% of those app users completed all four components of the study (*n* = 29; “study completers”). Similarly, 52% (*n* = 83) of consenting Pre-Lockdown participants were app users and 61% of those app users were study completers (*n* = 51).

As shown in Table 1, Fifty-five percent of app users were poor sleepers (ISI score > 10). Most app users (92.9%) were not seeing a health professional for a sleep problem and 78.6% were not seeing a health professional for a mental health disorder. In the Lockdown group, study completers did not differ from app user dropouts on age (*t*(48)=-.72, *p*=.478), sex (χ^2^(2) = 5.57, *p*=.062), ethnicity (χ^2^(6) = 7.51, *p*=.186), or ISI score (*t*(54)=-.09, *p*=.931). Among study completers, the Pre-Lockdown and Lockdown groups did not differ on age (*t*(71)=-.328, *p*=.744), sex (χ^2^(2)=.258, *p*=.612), ethnicity (χ^2^(6) = 10.24, *p*=.115), proportion of adolescents/young adults (χ^2^(1)=.759, *p*=.384), ISI score (*t*(78) = 1.14, *p*=.258), or proportion of good/poor sleepers (χ^2^(1) = 2.50, *p*=.114).

### Sleep before and during COVID-19 lockdowns

Sixty-five Pre-Lockdown participants and 40 Lockdown participants completed at least 7 days of sleep diaries for analysis. Pre-Lockdown (*M* = 12.35) and Lockdown (*M* = 12.92) participants did not differ in the number of sleep diary days (*p*=.138). Group differences in sleep at baseline are presented in Table 2. Participants in the Pre-Lockdown condition slept less than the recommended amount of sleep for adolescents and young adults (8–10 hours), and participants in the Lockdown condition spent significantly more time in bed. Compared to Pre-Lockdown participants, Lockdown participants reported a moderate but not statistically significant increase sleep duration, and they also reported spending more time awake in bed. Lockdown participants rose later than Pre-Lockdown participants and had significantly less variability in their rise time over the baseline period. SOL, WASO, SE, mean bedtime, and bedtime variability did not differ between the two groups. The two groups did not differ in the number of sleep diary days or self-reported insomnia severity.

**Table 2.** Differences in sleep before and during COVID-19 lockdowns among app users.

Variable	Pre-Lockdown (*N* = 65)	Lockdown (*N* = 40)	Benjamini-Hochberg adjusted *p*-value	Cohen’s *d*
Bedtime variability	4.34 (2.64)	3.32 (2.47)	.074	.399
Mean bedtime	23:57 (1:23)	23:20 (1:27)	.074	.429
Rise time variability	5.16 (3.36)	3.32 (2.20)	.**005**	.648
Mean rise time	8:46 (1:09)	9:34 (1:26)	.**020**	−.609
SOL	.44 (.39)	.52 (.47)	.377	−.185
WASO	.19 (.25)	.33 (.42)	.074	−.405
TIB	8.30 (.91)	9.32 (.76)	.**001**	−1.20
TST	7.29 (.91)	8.43 (3.91)	.098	−.406
TWT	1.03 (.55)	1.48 (.88)	.**016**	.613
SE	87% (7%)	85% (9%)	.128	.248

The false discovery rate was set at .05. Using the Benjamini-Hochberg procedure, each *p*-value is rank ordered from smallest to largest and evaluated in relation to its own corrected critical value, which is calculated using the following: (*i*/*m*)*q*, where i is the rank, *m* is the total number of tests, and *q* is the false discovery rate (in this case, .05).

### Sleep-related goal setting and behaviour change

#### Goal setting and tip use

Thirty-two Lockdown app users (57.1% of all Lockdown app users) and 57 Pre-Lockdown app users (68.7% of all Pre-Lockdown app users) made it to goal setting. Twenty-five Lockdown participants (78.1%) and 46 Pre-Lockdown participants (80.7%) set one or more goals to improve their sleep, while seven (21.9%) did not set goals. Lockdown participants set on average 1.53 goals (*SD* = 1.19; range: 0–5) and Pre-Lockdown participants set 1.54 goals (*SD* = 1.12; range: 0–4). Frequency of goals and tips are presented in Table 3.

**Table 3.** Frequency of goals and tips before and during COVID-19 lockdowns.

	Pre-Lockdown (*n* = 57)	Lockdown (*n* = 32)
**Goals**	***N*****(%)**	
Limit TIB to 8.5-10.5 hours	11 (19.3%)	14 (43.8%)
Reduce social jet lag	34 (59.6%)	11 (34.4%)
Reduce lingering in bed in the morning	16 (28.1%)	8 (25.0%)
Reduce sleep-interfering substance use	8 (14.0%)	6 (18.8%)
Reduce naps	17 (29.8%)	5 (15.6%)
Manage sleepiness	4 (7.0%)	3 (9.4%)
**Tips**	***N*****(%)**	
Difficulty winding down	24 (42.1%)	11 (34.4%)
Fatigue	13 (22.8%)	11 (34.4%)
Difficulty getting up	13 (22.8%)	5 (15.6%)
Being a night owl	17 (29.8%)	5 (15.6%)
Trouble staying awake	5 (8.8%)	3 (9.4%)

Among Lockdown participants, the most common goal was to limit TIB to 8.5–10.5 hours. Most participants who set this goal (*n* = 9) wanted to keep their TIB within this range on *both* weeknights and weekends. The remaining participants set a goal to restrict TIB to this range only on school nights. Participants also frequently set a goal to reduce social jet lag, most commonly to less than 2 hours, by keeping more consistent bedtimes and rise times throughout the week. These were followed by setting goals to target lingering in bed in the morning, reduce sleep-interfering substance use, reduce naps, and manage sleepiness. Lockdown participants most frequently accessed tips on “Difficulty Winding Down” and “Fatigue”, followed by “Difficulty Getting Up”, “Being a Night Owl”, and “Trouble Staying Awake” to help with achieving their goals. Pre-Lockdown participants focused on different goals. They most frequently set a goal to reduce jet lag, followed by reducing naps, targeting lingering in bed in the morning, limiting time in bed, and managing sleepiness. They also most frequently accessed “Difficulty Winding Down”, followed by “Being a Night Owl”, “Fatigue”, “Difficulty Getting Up”, and “Trouble Staying Awake”. No participants accessed the “Sleep Drunkenness” tip in either group.

Lockdown participants differed from Pre-Lockdown participants in that they less frequently set a goal to reduce jet lag, χ^2^(1) = 6.69, *p*=.010, and more frequently set a goal to restrict TIB, χ^2^(1) = 7.82, *p*=.005 (see Table 3). There were no differences in goal setting for morning lingering in bed (χ^2^(1)=.071, *p*=.791), sleepiness (χ^2^(1)=.177, *p*=.674), naps (χ^2^(1) = 2.09, *p*=.148), and sleep-interfering substance use (χ^2^(1)=.155, *p*=.694). The groups did not differ in their frequency of accessing tips on being a night owl (χ^2^(1) = 2.09, *p*=.148), difficulty winding down at night (χ^2^(1)=.426, *p*=.514), difficulty getting up in the morning (χ^2^(1)=.594, *p*=.441), sleep drunkenness (0% in both waves), daytime sleepiness (χ^2^(1)=.014, *p*=.904), or fatigue (χ^2^(1) = 2.03, *p*=.154).

#### Sleep-related behaviour change

Table 4 presents the 2 × 2 linear mixed models assessing sleep-related behaviours by group, time, and their interaction, as well as the means and standard errors for both groups at each timepoint. Significant group differences were observed in bedtime and rise time variability (indices of social jet lag), and morning lingering in bed. Compared to Pre-Lockdown, Lockdown participants had significantly less variable sleep schedules and spent more time lingering in bed in the morning across both time points. Morning lingering also decreased from baseline to post-feedback, averaged across both groups. Significant group × time interactions were observed for TIB and naps. Simple effects revealed that within Pre-Lockdown group, TIB increased from baseline to post-feedback (*B*=.022, *SE*=.09, *p*=.018), whereas TIB did not change significantly for participants in the Lockdown group (B=−.23, *SE*=.12, *p*=.053). In addition, participants in the Pre-Lockdown group significantly reduced their naps from baseline to post-feedback (*B*=-.36, *SE*=.10, *p* < .001), but there was no change in the Lockdown group (*B*=.02, *SE*=.13, *p*=.883).

**Table 4.** Sleep-related behaviour change after goal setting.

				Pre-Lockdown		Lockdown	
				Baseline	Post-Feedback	Baseline	Post-Feedback
Variable		*B (SE)*	*p*	*M (SD)*			
Bedtime variability (hours)^a^				4.34 (2.64)	4.15 (2.17)	3.32 (2.47)	2.72 (2.19)
	Group	−.38 (.12)	.**003**				
	Time	−.07 (.07)	.269				
	Group × time	−.04 (.13)	.770				
Rise time variability (hours)^b^				5.16 (3.36)	4.86 (2.55)	3.32 (2.20)	2.37 (1.23)
	Group	−.49 (.10)	**<.001**				
	Time	−.08 (.05)	.164				
	Group × time	−.16 (.11)	.129				
Time in bed (hours)^c^				9.32 (0.76)	8.30 (.91)	8.91 (.59)	8.43 (.84)
	Group	.77 (.14)	**<.001**				
	Time	−.01 (.08)	.934				
	Group × time	−.46 (.15)	.**003**				
Morning lingering (hours)^a^				.39 (.29)	.35 0.29)	.62 (.40)	.50 (.39)
	Group	.13 (.06)	.**025**				
	Time	−.05 (.02)	.**022**				
	Group × time	−.01 (.05)	.890				
Naps (hours)^b,*^				.46 (1.12)	.29 (.54)	.17 (.29)	.12 (.18)
	Group	−.47 (.20)	.**025**				
	Time	−.17 (.08)	.**042**				
	Group × time	.38 (.16)	.**024**				
Sleep-interfering substance use (proportion of days)^b,*^				.16 (.25)	.13 (.23)	.20 (.30)	.20 (.31)
	Group	.10 (.17)	.543				
	Time	−.02 (.06)	.790				
	Group × time	.04 (.12)	.715				

^a^Square root transformed.

^a^Log(10) transformed.

^b^Excluded *n* = 2 outliers.

*Due to the presence of 0 values, a constant was added to each observation to use the log(10) function.

Bolded values are *p* < .05

### Changes in sleep from baseline to post-feedback

To examine the impact of these behaviour changes, we explored whether sleep improved from baseline to post-feedback before and during COVID-19 lockdowns. Results of the linear mixed models and group means at all timepoints are presented in Table 5. SOL, WASO, SE, and ISI all significantly decreased from baseline to post-feedback in both groups. WASO was also significantly greater in the Lockdown group across both timepoints. TST demonstrated a significant group × time interaction. Among Pre-Lockdown participants, TST increased from baseline to post-feedback (*B*=.36, *SE*=.09, *p* < .001), yet there were no significant changes in TST for the Lockdown group (*B*=.04, *SE*=.12, *p*=.704). Mean bedtime also demonstrated a significant group × time interaction, such that the Lockdown group had an earlier bedtime than the Pre-Lockdown group at baseline (*B*=-.87, *SE*=.34, *p*=.011) but the groups did not differ post-feedback (*B*=-.48, *SE*=.34, *p*=.156).

**Table 5.** Changes in sleep indices from baseline to post-feedback before and during COVID-19 lockdowns.

				Pre-Lockdown		Lockdown	
				Baseline	Post-Feedback	Baseline	Post-Feedback
Variable		*B (SE)*	*p*	*M (SD)*			
SOL^a^				.44 (.39)	.31 (.30)	.52 (.47)	.37 (.24)
	Group	.16 (.12)	.184				
	Time	−.16 (.07)	.**030**				
	Group × time	.01 (.09)	.326				
WASO^a^				.19 (.25)	.15 (.23)	.33 (.42)	.20 (.28)
	Group	.38 (.18)	.**038**				
	Time	−.26 (.08)	.**001**				
	Group × time	−.20 (.15)	.203				
TST^b^				7.29 (.91)	7.55 (.80)	7.82 (.90)	7.84 (.59)
	Group	.38 (.17)	.**030**				
	Time	.20 (.07)	.**007**				
	Group × time	−.32 (.14)	.**034**				
SE				.87 (.07)	.90 (.07)	.85 (.09)	.88 (.07)
	Group	−.03 (.01)	.063				
	Time	.03 (.01)	**<.001**				
	Group × time	.00 (.01)	.813				
Mean bedtime				23.95 (1.39)	23.85 (1.61)	23.34 (1.45)	23.34 (1.27)
	Group	−.68 (.32)	.**039**				
	Time	.08 (.09)	.374				
	Group × time	.39 (.19)	.**040**				
Mean wake time				8.38 (1.16)	8.57 (1.62)	9.02 (1.39)	8.7 (1.24)
	Group	.24 (.29)	.401				
	Time	.11 (.11)	.312				
	Group × time	−.32 (.22)	.152				
ISI				12.17 (4.67)	8.24 (3.95)	10.22 (5.22)	6.93 (3.37)
	Group	−1.29 (.89)	.151				
	Time	−3.43 (.46)	**<.001**				
	Group × time	−.04 (.92)	.968				

ISI = Insomnia Severity Index, SE = Sleep Efficiency, SOL = Sleep Onset Latency, TST = Total Sleep Time, WASO = Wake After Sleep Onset.

## Discussion

We prospectively examined unconstrained sleep in AYAs reporting sleep dissatisfaction during COVID-19 lockdowns in Canada and compared their sleep and sleep behaviour changes to participants before COVID-19 lockdowns. At baseline, Lockdown participants spent more time in bed and their sleep duration was close to the recommended 8 to 10 hours (Paruthi et al., 2016), whereas Pre-Lockdown participants were in bed for significantly less time and their sleep duration was just over 7 hours. Using 2 weeks of prospective sleep monitoring, we replicated findings of increased self-reported TIB and TST in adolescents before and during COVID-19 lockdowns in the United States and Brazil (Becker et al., 2021; Dias Genta et al., 2021). Among both groups, insomnia indices were in the healthy range (i.e., SOL and WASO <30 min; SE between 85–90%). Thus, our prospective results indicate that AYAs were afforded more sleep opportunity during COVID-19 lockdowns, their sleep duration increased, and they demonstrated healthy sleep indices.

Sleep schedules also differed during lockdowns. Consistent with survey and qualitative data collected during COVID-19 lockdowns indicating that AYA’s bedtimes and rise times shifted later (Cellini et al., 2020; Gruber et al., 2020), our study found that Lockdown participants rose later in the morning (*M* = 9:34) than Pre-Lockdown participants (*M* = 8:46). This later rise time could account for the increase in TWT during lockdowns, but also the moderate increase in sleep duration. Consistent with previous research in university students during lockdowns (Wright et al., 2020), Lockdown participants also had significantly less variable rise times. We do not have data to support that school/work started later; presumably, *both* groups anchored their rise times to school/work start times during weekdays, but there were likely less discrepancies in schedules between weekdays and weekends during lockdowns due to reduced social and extracurricular activities. Our findings using prospective sleep monitoring largely comport with previous studies reporting reductions in social jet lag and daytime sleepiness, and increases in TIB, self-reported TST, sleep quality, and sleep regularity in AYAs during lockdowns (Becker et al., 2021; Dias Genta et al., 2021; Wright et al., 2020). Many are advocating for later school start times in North America (Wheaton et al., 2016), and evidence of improved sleep during lockdown may support this idea. Moreover, concerns that later rise times (and school start times) would result in later bedtimes were not supported. This suggests that participants did not delay their bedtimes (e.g., by increasing screen time) when afforded the chance, and instead slept longer.

This study also provided a unique glimpse into whether behaviours differed during COVID-19 lockdowns, and whether self-management is effective when AYAs have more control over their schedules. A similar proportion of Pre-Lockdown and Lockdown participants who created an account made it to the goal setting stage, and over half of app users used DOZE as directed for 4 weeks (until the end of the goal-directed behaviour change period). The groups differed in what goals they set based on their sleep feedback. Relative to Pre-Lockdown, Lockdown participants had more regular sleep schedules and less frequently set goals to reduce jet lag, and most commonly received feedback (and set a goal) to decrease TIB. App use was associated with significant behaviour change. Post-feedback, the Pre-Lockdown group *increased* their TIB but the Lockdown group’s reduction in TIB was not significant; at post-feedback, both groups spent similar amounts of TIB. Additionally, nearly one-third of Pre-Lockdown participants set a goal to reduce naps, and nap duration significantly reduced in this group. The frequency of setting other goals were the same across both groups, and both groups equally accessed the sleep tips to help them achieve their goals. Our data support that Lockdown participants made smaller behaviour changes than their Pre-Lockdown counterparts; it is probably that this is because they were sleeping better at baseline, resulting in less motivation (or need) to make larger changes.

Importantly, in this nonclinical sample, app use across both groups was associated with small improvements in sleep indices and self-report measures of insomnia. This is important because studies link COVID-19 lockdowns with increased self-reported insomnia, fatigue, anxiety, and depression among AYAs (Zhou et al., 2020), and rising levels of stress, depression, and anxiety have negatively influenced AYA sleep (Amaral et al., 2018). DOZE was developed to increase accessibility and address the low rate of treatment seeking in AYAs. Our findings support that free, user-designed apps can be a helpful tool for increasing access to sleep solutions for AYAs, and AYAs flexibly use these tools under variable circumstances. As a transdiagnostic self-management tool, DOZE is designed to be used by AYAs with diverse sleep needs and across the spectrum of severity to promote healthier sleep in this underserved group.

Our study has possible limitations. Analyses are likely underpowered due to our small sample size, with differences of medium effect size not reaching statistical significance. The number of statistical tests can increase experiment-wise error, which we controlled for using the Benjamini-Hochberg false discovery rate. Many consenting participants did not complete 4 weeks of app use, and the sample that remained may be highly motivated. The dropout rate in Lockdown was similar to Pre-Lockdown (Carmona et al., 2021). While the high dropout rate during various phases of the study (most notably during baseline questionnaire completion) is a limitation, the reported levels of dropout may also be reasonable for a nonclinical sample that may be less motivated for behaviour change. However, our data suggest that participants who used the app as intended began to work on the goals they set on the app. We did not collect data on participants’ geographic location in Canada or the severity of their province’s lockdowns, physical activity, social media use, and pandemic stress, and therefore cannot rule out their influences on sleep differences before and during lockdowns. It is important for future studies to collect data on AYAs’ educational/occupational status and how lockdowns changed their schedules to clarify how policy changes (e.g., school start times) can influence sleep schedules.

## Conclusions

COVID-19 lockdowns provided opportunities for AYAs to adjust their sleep health behaviours. Relative to a Pre-Lockdown cohort, AYAs during lockdowns could wake up later, sleep longer, and maintain less variable sleep schedules. This may be a product of decreased scheduling demands, such as academic, extracurricular, and social pressures. Offering a free self-management app to AYAs can help them improve their sleep in and out of lockdowns. COVID-19 is an ongoing public health concern and we hope that lockdowns will be unnecessary in the future, but we have learned valuable lessons: there may be opportunities to improve sleep, and evidence-based self-management apps fill an important void in improving sleep health in this group.

## Data Availability

The data that support the findings of this study are available from the corresponding author upon reasonable request.
